# PP2Acα promotes macrophage accumulation and activation to exacerbate tubular cell death and kidney fibrosis through activating Rap1 and TNFα production

**DOI:** 10.1038/s41418-021-00780-5

**Published:** 2021-05-01

**Authors:** Yan Liang, Xiaoli Sun, Mingjie Wang, Qingmiao Lu, Mengru Gu, Lu Zhou, Qing Hou, Mengzhu Tan, Sudan Wang, Xian Xue, Chunsun Dai

**Affiliations:** 1 Center for Kidney Disease, the 2nd Affiliated Hospital, Nanjing Medical University, Nanjing, Jiangsu China; 2 Department of Clinical Genetics, the 2nd Affiliated Hospital, Nanjing Medical University, Nanjing, Jiangsu China

**Keywords:** Cell biology, Inflammation

## Abstract

Macrophage accumulation and activation play an essential role in kidney fibrosis; however, the underlying mechanisms remain to be explored. By analyzing the kidney tissues from patients and animal models with kidney fibrosis, we found a large induction of PP2Acα in macrophages. We then generated a mouse model with inducible macrophage ablation of PP2Acα. The knockouts developed less renal fibrosis, macrophage accumulation, or tubular cell death after unilateral ureter obstruction or ischemic reperfusion injury compared to control littermates. In cultured macrophages, PP2Acα deficiency resulted in decreased cell motility by inhibiting Rap1 activity. Moreover, co-culture of PP2Acα^−/−^ macrophages with tubular cells resulted in less tubular cell death attributed to downregulated Stat6-mediated tumor necrosis factor α (TNFα) production in macrophages. Together, this study demonstrates that PP2Acα promotes macrophage accumulation and activation, hence accelerates tubular cell death and kidney fibrosis through regulating Rap1 activation and TNFα production.

## Introduction

Chronic kidney disease (CKD), defined as reduced glomerular filtration rate, increased urinary albumin excretion, or both, is becoming an increasing public health issue [1]. Regardless of the initial causes of CKD, interstitial fibrosis is the common pathologic characteristic and highly correlated with the long-term prognosis of CKD patients. Multiple events including inflammation, fibroblast activation, tubular atrophy, and microvascular rarefaction contribute to renal interstitial fibrosis [2]. Among them, renal fibrosis is always preceded and accompanied by the interstitial accumulation of various types of inflammatory cells, including macrophage, lymphocyte, mast cell, and dendritic cell [3, 4]. As the major mediator of inflammatory responses, macrophage accumulation is highly involved in the process of renal injury and repair in many types of experimental and human renal disease [5–9].

Upon recruitment into the injured kidneys, macrophages are activated and differentiated into two distinct subsets named M1 and M2 in response to different cues [10, 11]. Activated macrophages may produce a wide range of pro-inflammatory molecules, including tumor necrosis factor α (TNFα), interleukin-1β (IL-1β), IL-12, and inducible nitric oxide synthase [12]. These cytokines alter the renal microenvironment and regulate the fate of the cells such as tubular cell and determine the outcome of the diseased kidneys [5, 10, 13]. Therefore, deciphering the mechanisms regulating macrophage activation and the interplay between macrophages and tubular cells is necessary for retarding kidney fibrosis.

There are multiple subunits of each of the PP2A components including scaffold A (PP2A_A_), regulatory B (PP2A_B_), and catalytic C (PP2A_C_) [14, 15]. PP2A has been reported to participate in the pathogenesis of many types of disease such as cancer, neurodegenerative diseases, systemic lupus erythematosus as well as kidney diseases [15, 16]. Zhong et al. reported that specific deletion of PP2A in podocytes exacerbated diabetic kidney disease in mice [17]. Sun et al. reported that mice with myeloid-specific deletion of PP2Acα developed more exuberant fibrosis, elevated cytokine responses, and chronic myeloid inflammation in response to bleomycin [18]. However, the role and mechanisms for PP2Acα in regulating macrophage activation and kidney fibrosis remain to be determined.

In this study, we report that PP2Acα was upregulated in macrophages from the fibrotic kidneys. Macrophage-specific ablation of PP2Acα in mice attenuated inflammatory cell accumulation, tubular cell death, and renal fibrosis after unilateral ureteral occlusion (UUO) or ischemia reperfusion injury (IRI). In cultured macrophages, PP2Acα depletion decreased macrophage migration and tubular cell death by inhibiting Rap1 and Stat6-stimulated TNFα production, respectively.

## Results

### The induction of PP2Acα in macrophages from mouse and human fibrotic kidneys

**Fig. 1:**
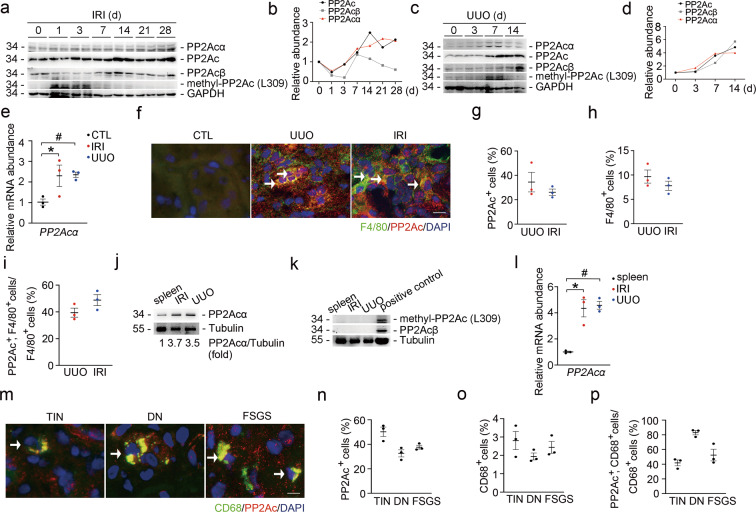
The upregulation of PP2Acα in macrophages from mouse and human fibrotic kidneys. **a**, **b** Western blot assay (**a**) and graphic presentation (**b**) showing the abundance of PP2Ac, PP2Acα, PP2Acβ, and methyl-PP2Ac (L309) in the kidneys after IRI. **c**, **d** Western blot assay (**c**) and graphic presentation (**b**) showing the abundance of PP2Ac, PP2Acα, PP2Acβ, and PP2Ac (methyl L309) in the kidneys after UUO. **e** Real-time qRT-PCR analysis showing the mRNA abundance of PP2Acα in kidneys. *p < 0.05, *n* = 3. ^#^*p* < 0.05, *n* = 3. Data are presented as means ± SEM. **f** Representative immune staining images showing the expression of PP2Ac in F4/80-positive macrophages within the fibrotic kidneys at day 14 or day 28 after UUO or IRI, respectively. White arrows indicate co-staining-positive cells. Scale bar, 5 μm. **g** Quantitative analysis for PP2Ac-staining-positive cells in mouse kidney tissues. *n* = 3. Data are presented as means ± SEM. **h** Quantitative analysis for F4/80-staining-positive macrophages in mouse kidney tissues. *n* = 3. Data are presented as means ± SEM. **i** Quantitative analysis for PP2Ac and F4/80 double-staining-positive cells in mice kidney tissues. *n* = 3. Data are presented as means ± SEM. **j** Western blot assay showing the induction of PP2Acα in macrophages sorted from UUO or IRI kidneys with CD115 microbeads. **k** Western blot assay showing PP2A_C_β and methyl-PP2Ac (L309) in macrophages sorted from spleen, UUO, or IRI kidneys with CD115 microbeads. Mouse kidney lysate was utilized as positive control. **l** Real-time qRT-PCR analysis showing the mRNA abundance of PP2Acα in macrophages sorted from spleen, UUO, or IRI with CD115 microbeads. *p < 0.05, *n* = 3. ^#^*p* < 0.05, *n* = 3. Data are presented as means ± SEM. **m** Representative immune staining images showing the expression of PP2Ac in CD68-staining-positive macrophages within human fibrotic kidneys. White arrows indicate co-staining-positive cells. Scale bar, 5 μm. **n** Quantitative analysis for PP2Ac-staining-positive cells in human kidney tissues, *n* = 3. Data are presented as means ± SEM. **o** Quantitative analysis for CD68-staining-positive cells in human kidney tissues. *n* = 3. Data are presented as means ± SEM. **p** Quantitative analysis for PP2Ac and CD68 double-staining-positive cells in human kidney tissues. *n* = 3. Data are presented as means ± SEM.

To explore the role for PP2Aca in kidney fibrosis, male CD1 mice were operated with UUO or IRI. Western blot analyses revealed that PP2Ac and PP2Acα abundance was markedly increased at day 7 after UUO and day 14 after IRI, respectively. PP2Acβ abundance was increased at day 14 after UUO and IRI. The methyl-PP2Ac (L309) abundance was increased at days 1 and 3 after IRI and at day 7 after UUO (Fig. 1a–d). The mRNA abundance of PP2Acα in the fibrotic kidneys was significantly increased compared to the control kidneys (Fig. 1e). Co-staining of F4/80 and PP2Ac showed upregulation of PP2Ac in macrophages from the fibrotic kidneys (Fig. 1f). About 20 and 50% of kidney cells were stained positive for PP2Ac (Fig. 1g), whereas about 6 and 12% of kidney cells were stained positive for F4/80 in UUO and IRI kidneys, respectively (Fig. 1h). Among F4/80-staining-positive macrophages, about 34 and 55% of them were PP2Ac staining positive in UUO and IRI kidneys, respectively (Fig. 1i). We sorted macrophages from spleen and the fibrotic kidneys with CD115 microbeads. Western blot assay showed that PP2Acα abundance in macrophages from UUO- and IRI-induced fibrotic kidneys was increased more than three folds to those from spleen (Fig. 1j). However, PP2Acβ and methyl-PP2Ac (L309) were not detected in the sorted macrophages from either spleen or the fibrotic kidneys (Fig. 1k). The mRNA abundance of PP2Acα in macrophages from the fibrotic kidneys was significantly increased compared to those from the spleen (Fig. 1l). We also stained kidney biopsies from patients with tubular interstitial nephritis (TIN), diabetic nephrology (DN), and focal segmental glomerular sclerosis (FSGS) with antibodies against CD68 and PP2Ac, respectively. About 35–55% of kidney cells were stained positive for PP2Ac (Fig. 1n). About 2–3.5% of kidney cells were stained positive for CD68 (Fig. 1o). About 33–67% of CD68 positive macrophages were stained positive for PP2Ac (Fig. 1p). Collectively, these data indicate that PP2Acα expression is upregulated in macrophages from both mouse and human fibrotic kidneys.

### Ablation of PP2Acα in macrophages ameliorates kidney fibrosis

**Fig. 2:**
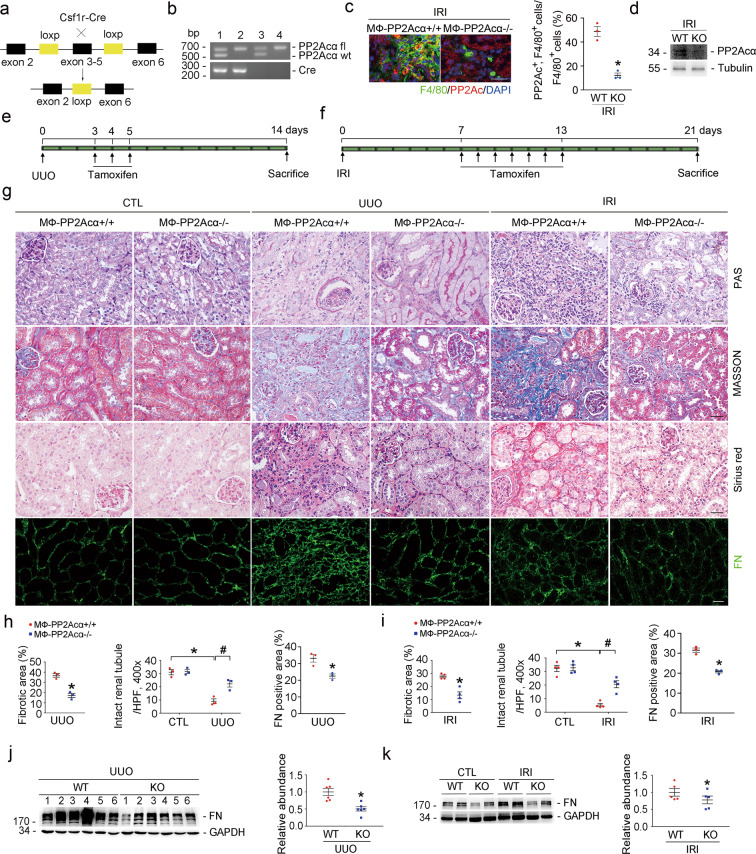
Ablation of PP2Acα in macrophages ameliorates kidney fibrosis. **a** Schematic diagram showing the ablation of PP2Acα in macrophages. **b** PCR analysis for genotyping the mice. Lane 1: Csf1r-Cre^+/−^, PP2Acα^fl/wt^; Lane 2: Csf1r-Cre^+/−^, PP2Acα^fl/fl^; Lane 3: Csf1r-Cre^−/−^, PP2Acα^fl/wt^; Lane 4: Csf1r-Cre^−/−^, PP2Acα^fl/fl^. **c** Representative micrographs and quantitative analysis showing the ablation of PP2Acα in F4/80-positive macrophages in IRI kidneys from Csf1r-Cre^+/−^, PP2Acα^fl/fl^ mice. Scale bar, 20 μm. **d** Western blot assay for PP2A_C_α in macrophages sorted from IRI kidneys of MФ-PP2Acα^+/+^ and MФ-PP2Acα^−/−^ mice with CD115 microbeads. **e**, **f** Strategies for the surgery and tamoxifen administration in Csf1r-Cre^+/−^, PP2Acα^fl/fl^ mice and their control littermates. **g** Representative micrographs for PAS, Masson, Sirius red, and FN staining in kidney tissues. Scale bar, 20 μm. **h** Graphic presentation showing the fibrotic area, intact renal tubule, and FN-staining-positive area in UUO kidney tissues among groups as indicated. *p < 0.05, *n* = 3. ^#^*p* < 0.05, *n* = 3. Data are presented as means ± SEM. **i** Graphic presentation showing the fibrotic area, intact renal tubule, and FN-staining-positive area in IRI kidney tissues among groups as indicated. *p < 0.05, *n* = 4. ^#^*p* < 0.05, *n* = 4. Data are presented as means ± SEM. **j** Western blot analyses (left) and quantitative analysis (right) for FN in UUO kidneys from the knockouts and control littermates. Numbers 1–6 indicate each individual animal within a given group. *p < 0.05, *n* = 6. Data are presented as means ± SEM. **k** Western blot analyses (left) and quantitative analysis (right) for FN in CTL and IRI kidneys from different groups as indicated. *p < 0.05, *n* = 5. Data are presented as means ± SEM.

To investigate the role for macrophage PP2Acα induction in kidney fibrosis, we generated a mouse model with inducible macrophage PP2Acα ablation by crossbreeding Csf1r-Cre and PP2Acα^fl/fl^ mice (Fig. 2a, b). Csf1r-Cre^+/−^, PP2Acα^fl/fl^ mice, and Csf1r-Cre^−/−^, PP2Acα^fl/fl^ mice were subjected to UUO or IRI, respectively, and tamoxifen was injected intraperitoneally as indicated (Fig. 2e, f). Immunofluorescence staining and western blot analyses showed successfully deletion of PP2Acα in macrophages from MФ-PP2Acα^−/−^ mice (Fig. 2c, d). In MФ-PP2Acα^+/+^ kidneys after UUO or IRI, remarkable tubular atrophy and interstitial extracellular matrix deposition were detected, which were markedly attenuated in the knockout kidneys (Fig. 2g). Notably, the integrity of renal tubule was largely preserved in the knockout fibrotic kidneys (Fig. 2h, i). In addition, both immunofluorescence staining (Fig. 2g–i) and western blot analyses (Fig. 2j, k) showed that FN protein abundance was largely reduced in MФ-PP2Acα^−/−^ kidneys compared to those in MФ-PP2Acα^+/+^ fibrotic kidneys. Together, these results suggest that deletion of PP2Acα in macrophages preserves tubular integrity and attenuates UUO- or IRI-induced kidney fibrosis.

### Ablation of PP2Acα in macrophages reduces inflammatory cell accumulation in the fibrotic kidneys

**Fig. 3:**
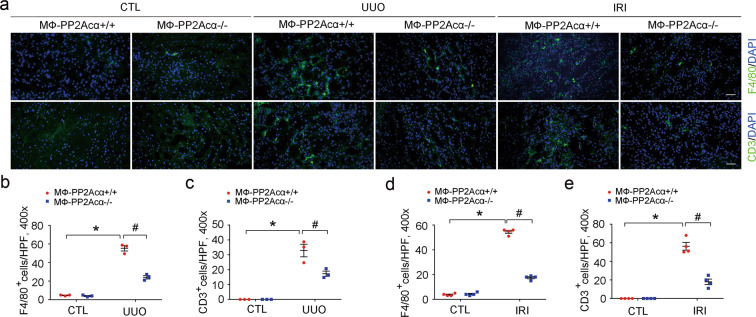
Ablation of PP2Acα in macrophages reduces inflammatory cell accumulation in the fibrotic kidneys. **a** Representative staining images for F4/80 and CD3 in UUO and IRI kidneys. Scale bar, 20 μm. **b**, **c** Quantitative analysis for F4/80-positive cells (**b**) and CD3-positive cells (**c**) in UUO kidney tissues. *p < 0.05, *n* = 3. ^#^*p* < 0.05, *n* = 3. Data are presented as means ± SEM. **d**, **e** Quantitative analysis for F4/80-positive cells (**d**) and CD3-positive cells (**e**) in IRI kidney tissues. *p < 0.05, *n* = 4. ^#^*p* < 0.05, *n* = 4. Data are presented as means ± SEM.

To explore the mechanisms for macrophage PP2Acα ablation in ameliorating kidney fibrosis, we examined the inflammatory cell accumulation within the fibrotic kidneys. In MФ-PP2Acα^+/+^ fibrotic kidneys, macrophage accumulation was largely enhanced, which was reduced about 55% in MФ-PP2Acα^−/−^ fibrotic kidneys (Fig. 3a, b, d). The T lymphocytes accumulation was also markedly attenuated in MФ-PP2Acα^−/−^ fibrotic kidneys (Fig. 3a, c, e). Thus, these data show that depletion of macrophage PP2Acα in mice reduces macrophage accumulation in the fibrotic kidneys.

### PP2Acα ablation inhibits macrophage migration by suppressing Rap1

**Fig. 4:**
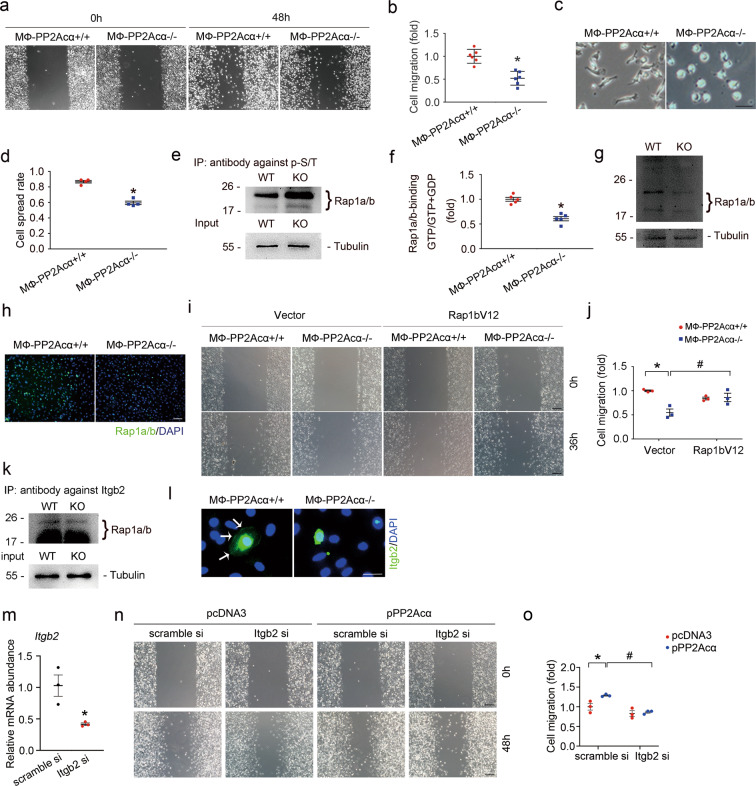
PP2Acα ablation inhibits macrophage migration by suppressing Rap1. **a**, **b** Representative images (**a**) and quantitative analysis (**b**) for wound healing test in cultured BMDMs. Scale bar, 100 μm. *p < 0.05, *n* = 6. Data are presented as means ± SEM. **c**, **d** Representative images (**c**) and quantitative analysis (**d**) for cell spreading in cultured BMDMs. Scale bar, 10 μm. *p < 0.05, *n* = 4. Data are presented as means ± SEM. **e** Immunoprecipitation assay showing the induction of serine/threonine phosphorylation of Rap1a/b in PP2Acα^−/−^ BMDMs. **f** Graphic presentation showing the activity of Rap1a/b in PP2Acα^+/+^ and PP2Acα^−/−^ BMDMs. *p < 0.05, *n* = 5. Data are presented as means ± SEM. **g** Western blot analyses showing the reduction of Rap1a/b in PP2Acα^−/−^ BMDMs. **h** Representative immune staining images for Rap1a/b in PP2Acα^+/+^ and PP2Acα^−/−^ BMDMs. Scale bar, 100 μm. **i**, **j** Representative images (**i**) and quantitative analysis (**j**) for wound healing test in cultured BMDMs from different groups as indicated. Scale bar, 100 μm. *p < 0.05, *n* = 3. ^#^*p* < 0.05, *n* = 3. Data are presented as means ± SEM. **k** Immunoprecipitation assay showing the binding of Rap1a/b and Itgb2 in BMDMs. **l** Representative immune staining images showing the clustering of Itgb2 in PP2Acα^+/+^ and PP2Acα^−/−^ BMDMs cultured on the plate coated with fibronectin. White arrows indicate Itgb2 clustering. Scale bar, 10 μm. **m** Real-time qRT-PCR analysis showing the mRNA abundance of *Itgb2* in scramble siRNA and Itgb2 siRNA-transfected BMDMs. *p < 0.05, *n* = 3. Data are presented as means ± SEM. **n**, **o** Representative images (**n**) and quantitative analysis (**o**) for wound healing test in cultured BMDMs from different groups as indicated. Scale bar, 100 μm. *p < 0.05, *n* = 3. ^#^*p* < 0.05, *n* = 3. Data are presented as means ± SEM.

The above data show that ablation of PP2Acα in macrophages led to less macrophage accumulation within the fibrotic kidneys. Macrophage accumulation was determined by cell proliferation, survival, and infiltration. Less than 1% of the macrophages were terminal deoxynucleotidyl transferase-mediated dUTP nick-end labeling (TUNEL)-staining positive in MФ-PP2Acα^+/+^ and MФ-PP2Acα^−/−^ fibrotic kidneys (Supplementary Fig. 2a). In cultured PP2Acα^−/−^ bone marrow-derived macrophages (BMDMs), macrophage proliferation was similar to those in PP2Acα^+/+^ BMDMs (Supplementary Fig. 2b, c). Therefore, these data suggest that less macrophage accumulation in MФ-PP2Acα^−/−^ fibrotic kidneys does not attribute to the alteration of macrophage survival or proliferation. Intriguingly, in the peripheral blood of MФ-PP2Acα^+/+^ mice, about 8% of the Ly6g negative cells were Ly6c- and CD11b-positive monocytes/macrophages, while only 5.5% of the cells were monocytes/macrophages in that of MФ-PP2Acα^−/−^ mice (~31% reduction) (Supplementary Fig. 3). Therefore, it is conceivable that less macrophage accumulation in MФ-PP2Acα^−/−^ fibrotic kidneys is due to diminished macrophage infiltration. To further prove that, we cultured BMDMs and found that macrophage migration and spreading were largely attenuated by PP2Acα depletion (Fig. 4a–d). We then explored the mechanisms regulating macrophage migration. Rap1 was reported to regulate integrin-mediated adhesion and spreading in various mammalian cell types. We wondered whether PP2Acα regulates macrophage migration through modulating Rap1 activity. In PP2Acα^−/−^ BMDMs, the serine/threonine phosphorylation of Rap1 was largely induced (Fig. 4e). Moreover, Rap1 protein abundance and activity were markedly reduced in PP2Acα^−/−^ BMDMs (Fig. 4f–h). Therefore, all data indicate Rap1 as the target of PP2Acα. To clarify the role for Rap1 in PP2Acα-regulated macrophage migration, we transfected PP2Acα^−/−^ BMDMs with Rap1bV12 (a dominant active Rap1b mutant) plasmid and the results showed that overexpression of Rap1bV12 largely restored macrophage motility in PP2Acα^−/−^ BMDMs, indicating the crucial role for Rap1b in mediating PP2Acα-regulated macrophage motility (Fig. 4i, j). It has been reported that Rap1 activity may be regulated by its guanine nucleotide exchange factors (GEFs) and GTPase-activating proteins (GAPs). In this study, we found that the protein abundance of Epac1, a cAMP responsive GEF, was not altered by PP2Acα deletion or overexpression (Supplementary Fig. 4a, b). In addition, knocking down Epac1 had little effect on PP2Acα-stimulated macrophage motility (Supplementary Fig. 4c–e). Cell adhesion and migration are tightly regulated by integrins. In BMDMs, PP2Acα deficiency resulted in decreased binding of Rap1 and integrin β2 (Itgb2) (Fig. 4k). To investigate whether PP2Acα deficiency affects Itgb2 activation in macrophages, we cultured BMDMs on the plate coated with fibronectin. Immunofluorescence staining results showed that PP2Acα deficiency reduced the clustering of Itgb2 (Fig. 4l). Furthermore, knocking down Itgb2 antagonized PP2Acα overexpression-stimulated cell motility (Fig. 4m–o), suggesting that PP2Acα regulates macrophage motility through modulating Rap1/integrin β2 pathway. Collectively, these data suggest that PP2Acα ablation reduces Rap1 protein abundance and activity to inhibit Itgb2-regulated macrophage migration and infiltration.

### Macrophage PP2Acα ablation facilitates tubular cell survival via downregulating Stat6-mediated TNFα production

**Fig. 5:**
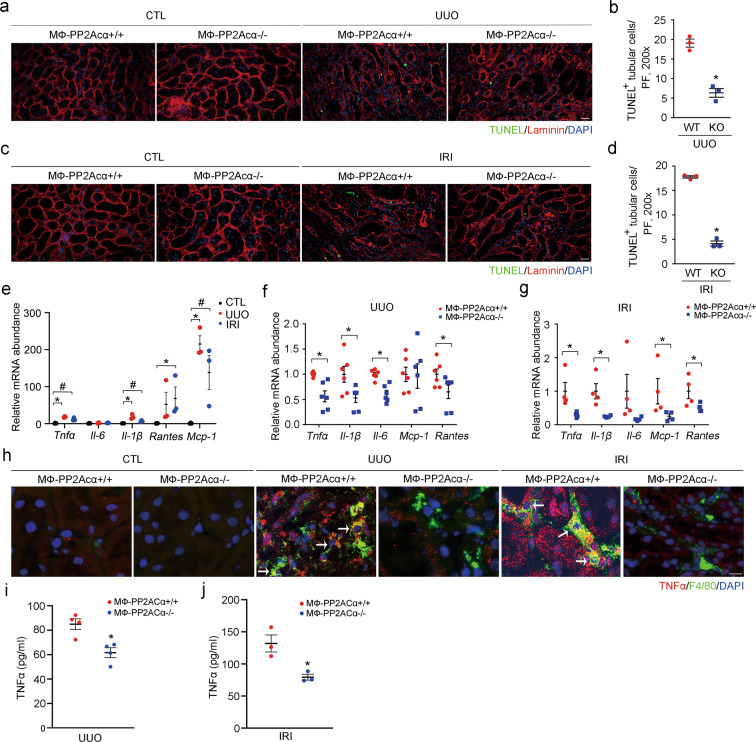
Macrophage PP2Acα ablation facilitates tubular cell survival. **a** Representative micrographs for TUNEL and laminin co-staining in UUO kidneys. Scale bar, 20 μm. **b** Quantitative analysis for TUNEL-positive tubular cells in UUO kidneys. *p < 0.05, *n* = 3. Data are presented as means ± SEM. **c** Representative micrographs for TUNEL and laminin co-staining in IRI kidneys. Scale bar, 20 μm. **d** Quantitative analysis for TUNEL-positive tubular cells in IRI kidneys. *p < 0.05, *n* = 3. Data are presented as means ± SEM. **e** Real-time qRT-PCR analysis showing the mRNA abundance of *Tnfα*, *Il-1β*, *Il-6*, *Mcp1*, and *Rantes* in the control (CTL) and fibrotic kidneys. *p < 0.05, *n* = 3. ^#^*p* < 0.05, *n* = 3. Data are presented as means ± SEM. **f** Real-time qRT-PCR analysis showing the mRNA abundance of *Tnfα*, *Il-1β*, *Il-6*, *Mcp1*, and *Rantes* in UUO kidneys from MФ-PP2Acα^+/+^ and MФ-PP2Acα^−/−^ mice. *p < 0.05, *n* = 6. Data are presented as means ± SEM. **g** Real-time qRT-PCR analysis showing the mRNA abundance of *Tnfα*, *Il-1β*, *Il-6*, *Mcp1*, and *Rantes* in IRI kidneys from MФ-PP2Acα^+/+^ and MФ-PP2Acα^−/−^ mice. *p < 0.05, *n* = 4. Data are presented as means ± SEM. **h** Representative immune staining images showing the induction of TNFα in F4/80-staining-positive macrophages within the kidneys after UUO or IRI among groups as indicated. White arrows indicate co-staining-positive macrophages. Scale bar, 10 μm. **i** ELISA assay for TNFα in UUO kidney tissues from MФ-PP2Acα^+/+^ and MФ-PP2Acα^−/−^ mice. *p < 0.05, *n* = 4. Data are presented as means ± SEM. **j** ELISA assay for TNFα in IRI kidney tissues from MФ-PP2Acα^+/+^ and MФ-PP2Acα^−/−^ mice. *p < 0.05, *n* = 3. Data are presented as means ± SEM.

In addition to reduced macrophage accumulation, the above data show that mice with macrophage PP2Acα ablation exhibited more intact tubule compared to control littermates. Therefore, it was predicted that ablation of PP2Acα in macrophages may also protect against tubular cell death after UUO or IRI. To determine that, we co-stained kidney tissues with laminin and TUNEL and the results showed that less tubular cell death was found in MФ-PP2Acα^−/−^ fibrotic kidneys compared to those in MФ-PP2Acα^+/+^ kidneys (Fig. 5a–d).

We then wanted to know what mediates the interplay between macrophages and tubular cells during kidney fibrosis. It had been reported that activated macrophages may secrete large amounts of inflammatory cytokines, including TNFα, IL-1β, and IL-6. Among them, TNFα could mediate obstruction-induced tubular cell apoptosis [19]. Hence, we predicted that macrophage PP2Acα ablation may downregulate TNFα production to facilitate tubular cell survival. The mRNA abundance of pro-inflammatory cytokines including *Tnfα*, *Il1β*, and *Mcp-1* was significantly upregulated in the fibrotic kidneys (Fig. 5e). In MФ-PP2Acα^+/+^ and MФ-PP2Acα^−/−^ kidneys, no significant difference was observed in the mRNA abundance of pro-inflammatory cytokines including *Tnfα*, *Il-6*, *Il-1β*, *Rantes*, and *Mcp-1* (Supplementary Fig. 5a). However, compared to those in MФ-PP2Acα^+/+^ kidneys after UUO or IRI, the mRNA abundance of most of the pro-inflammatory cytokines such as TNFα was much less in MФ-PP2Acα^−/−^ kidneys, respectively (Fig. 5f, g). We then co-stained kidney tissues with antibodies against F4/80 and TNFα. The results showed that TNFα could be detected in F4/80-positive macrophages, as well as in T lymphocytes and tubular cells of the fibrotic kidneys (Fig. 5h and Supplementary Fig. 5b, c). The abundance of TNFα in MФ-PP2Acα^−/−^ kidneys was largely decreased compared to that in MФ-PP2Acα^+/+^ kidneys after UUO or IRI, respectively (Fig. 5i, j). Notably, TNFα in macrophages from MФ-PP2Acα^−/−^ fibrotic kidneys was much less than that from MФ-PP2Acα^+/+^ kidneys (Fig. 5h).

**Fig. 6:**
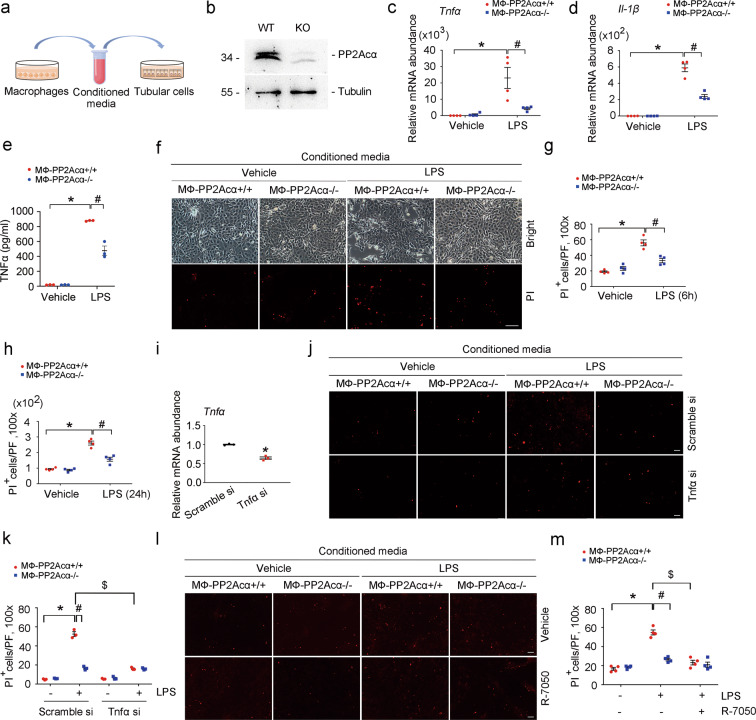
PP2Acα ablation protects against tubular cell death through inhibiting TNFα production in macrophages. **a** The co-culture system of macrophages and tubular cells. **b** Western blot analyses showing the ablation of PP2Acα in PP2Acα^−/−^ BMDMs. **c**, **d** Real-time qRT-PCR analysis showing the mRNA abundance of *Tnfα* (**c**) and *Il-1β* (**d**) in PP2Acα^+/+^ and PP2Acα^−/−^ BMDMs with or without LPS treatment. *p < 0.05, *n* = 4. ^#^*p* < 0.05, *n* = 4. Data are presented as means ± SEM. **e** ELISA analysis showing the protein abundance of TNFα in the cultural media of BMDMs with or without LPS treatment. *p < 0.05, *n* = 3. ^#^*p* < 0.05, *n* = 3. Data are presented as means ± SEM. **f** Representative images of bright field and PI staining of BMDMs. Scale bar, 100 μm. **g**, **h** Quantitative analyses of PI-staining-positive tubular cells at 6 h (**g**) and 24 h (**h**) after LPS treatment among groups as indicated. *p < 0.05, *n* = 4. ^#^*p* < 0.05, *n* = 4. Data are presented as means ± SEM. **i** Real-time qRT-PCR analysis showing the mRNA abundance of *Tnfα* in scramble siRNA and Tnfα siRNA-transfected BMDMs. *p < 0.05, *n* = 3. Data are presented as means ± SEM. **j**, **k** Representative PI staining images (**j**) and quantitative analyses (**k**) showing that the conditioned media (CM) of *Tnfα* siRNA-transfected macrophages caused less tubular cell death. Scale bar, 100 μm. *p < 0.05, *n* = 3. ^#^*p* < 0.05, *n* = 3. ^$^*p* < 0.05, *n* = 3. Data are presented as means ± SEM. **l**, **m** Representative PI staining images (**l**) and quantitative analysis (**m**) showing that R-7050 treatment could reduce tubular cell death. Scale bar, 100 μm. *p < 0.05, *n* = 4. ^#^*p* < 0.05, *n* = 4. ^$^*p* < 0.05, *n* = 4. Data are presented as means ± SEM.

To decipher the role for macrophage-derived TNFα in regulating tubular cell death, we employed a co-culture system of macrophages and tubular cells (Fig. 6a). BMDMs with PP2Acα deletion were generated (Fig. 6b). TNFα mRNA and protein expression was upregulated in PP2Acα^+/+^ BMDMs treated with LPS, which was much less in PP2Acα^−/−^ BMDMs (Fig. 6c, e). Next, BMDMs were treated with LPS for 6 h, then the cultural medium was changed with serum-free medium. Twelve hours later, conditioned media (CM) from macrophages was harvested to treat mouse primary cultured tubular cells. Propidium iodide (PI) staining showed that the CM from LPS-treated PP2Acα^+/+^ macrophages significantly promoted tubular cell death, whereas much less cell death was detected in those treated with CM from PP2Acα^−/−^ macrophages (Fig. 6f–h). To clarify the role for macrophage-derived TNFα in tubular cell death, we silenced macrophage TNFα expression with small interfering RNA (siRNA) transfection (Fig. 6i). As expected, knocking down macrophage TNFα expression significantly reduced tubular cell death caused by CM from LPS-treated PP2Acα^+/+^ macrophages (Fig. 6j, k). In addition, tubular cells were treated with R-7050 to block intracellular TNFR signaling. The results showed that R-7050 could significantly inhibit tubular cell death caused by CM from LPS-treated PP2Acα^+/+^ macrophages, while R-7050 could not further decrease cell death in tubular cells treated with CM from LPS-treated PP2Acα^−/−^ macrophages, indicating the essential role for TNFα in mediating macrophage-caused tubular cell death (Fig. 6l, m). Above all, it is concluded that macrophage PP2Acα ablation decreased TNFα expression, hence facilitated tubular cell survival.

**Fig. 7:**
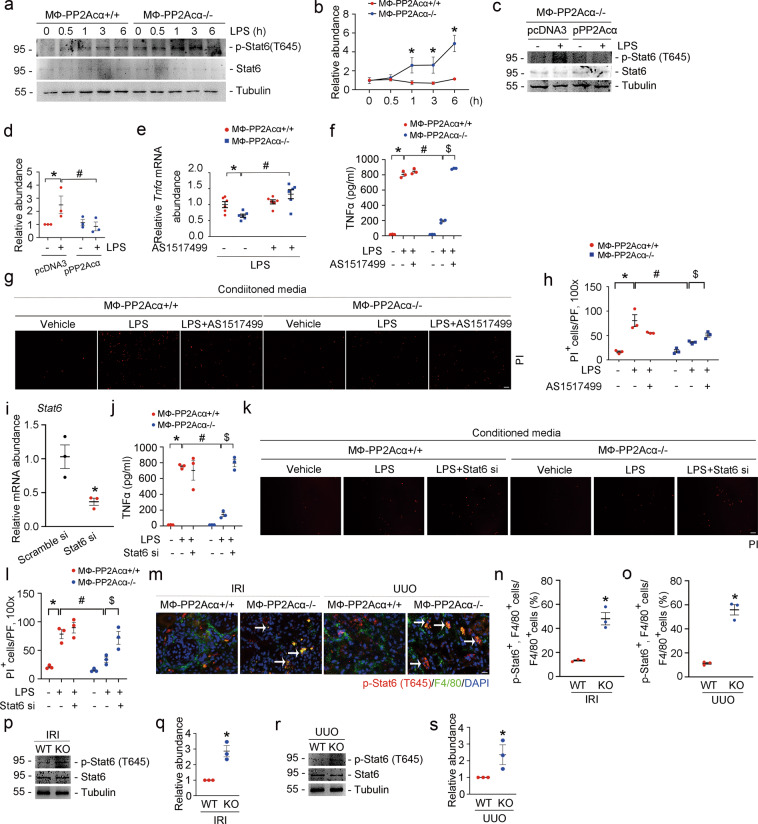
Macrophage PP2Acα ablation facilitates tubular cell survival via downregulating Stat6-mediated TNFα production. **a**, **b** Western blot assay (**a**) and semi-quantitative analysis (**b**) showing that deletion of PP2Acα enhanced LPS-stimulated Stat6 phosphorylation in BMDMs. *p < 0.05, *n* = 3. Data are presented as means ± SEM. **c**, **d** Western blot assay (**c**) and quantitative analysis (**d**) showing that overexpression of PP2Acα could inhibit Stat6 phosphorylation caused by PP2Acα ablation. *p < 0.05, *n* = 3. ^#^*p* < 0.05, *n* = 3. Data are presented as means ± SEM. **e** Real-time qRT-PCR analysis showing the mRNA abundance of *Tnfα* in BMDMs. *p < 0.05, *n* = 6. ^#^*p* < 0.05, *n* = 6. Data are presented as means ± SEM. **f** ELISA assay showing the protein abundance of TNFα in the cultural media of AS1517499-treated BMDMs. *p < 0.05, *n* = 3. ^#^*p* < 0.05, *n* = 3. ^$^*p* < 0.05, *n* = 3. Data are presented as means ± SEM. **g**, **h** Representative PI staining images (**g**) and quantitative analysis (**h**) showing that the conditioned media of AS1517499-treated macrophages caused more tubular cell death. Scale bar, 100 μm. *p < 0.05, *n* = 3. ^#^*p* < 0.05, *n* = 3. ^$^*p* < 0.05, *n* = 3. Data are presented as means ± SEM. **i** Real-time qRT-PCR analysis showing the mRNA abundance of *Stat6* in scramble siRNA and Stat6 siRNA-transfected BMDMs. *p < 0.05, *n* = 3. Data are presented as means ± SEM. **j** ELISA analysis showing the protein abundance of TNFα in the cultural media of Stat6 siRNA-transfected BMDMs. *p < 0.05, *n* = 3. ^#^*p* < 0.05, *n* = 3. ^$^*p* < 0.05, *n* = 3. Data are presented as means ± SEM. **k**, **l** Representative PI staining images (**k**) and quantitative analysis (**l**) showing that the conditioned media of Stat6 siRNA-transfected macrophages caused more tubular cell death. Scale bar, 100 μm. *p < 0.05, *n* = 3. ^#^*p* < 0.05, *n* = 3. ^$^*p* < 0.05, *n* = 3. Data are presented as means ± SEM. **m** Representative immune staining images showing the induction of p-Stat6 (T645) in F4/80-positive macrophages within MФ-PP2Acα^−/−^ fibrotic kidneys. White arrows indicate double-staining-positive cells. Scale bar, 10 μm. **n**, **o** Quantitative analysis for p-Stat6 (T645) and F4/80 double-positive cells in IRI (**n**) and UUO (**o**) kidney tissues. *p < 0.05, *n* = 3. Data are presented as means ± SEM. **p**, **q** Western blot analyses (**p**) and quantitative determination (**q**) showing that deletion of PP2Acα could induce the phosphorylation of Stat6 in macrophages sorted from MФ-PP2Acα^−/−^ IRI kidneys. *p < 0.05, *n* = 3. Data are presented as means ± SEM. **r**, **s** Western blot analyses (**r**) and quantitative determination (**s**) showing that deletion of PP2Acα could induce the phosphorylation of Stat6 in macrophages sorted from MФ-PP2Acα^−/−^ UUO kidneys. *p < 0.05, *n* = 3. Data are presented as means ± SEM.

Stat6 activation could prohibit macrophage M1 polarization by inhibiting Stat1 and Irf-5, which are essential for the production of pro-inflammatory cytokines. To explore whether Stat6 mediates the downregulation of TNFα in PP2Acα^−/−^ macrophages, we examined p-Stat6 (T645) in PP2Acα^−/−^ BMDMs and PP2Acα^+/+^ BMDMs. The results showed that p-Stat6 abundance was much more in PP2Acα^−/−^ BMDMs compared to PP2Acα^+/+^ BMDMs after LPS treatment (Fig. 7a, b). Moreover, overexpression of exogenous PP2Acα inhibited Stat6 phosphorylation caused by PP2Acα ablation (Fig. 7c, d). To further investigate whether PP2Acα deficiency inhibits TNFα expression via Stat6 activation, we treated PP2Acα^−/−^ BMDMs with AS1517499, a small molecule of Stat6 inhibitor. The results showed that AS1517499 could restore TNFα mRNA and protein expression in PP2Acα^−/−^ BMDMs (Fig. 7e, f). In addition, tubular cell death induced by CM from PP2Acα^−/−^ macrophages treated with AS1517499 was exacerbated, indicating the essential role for macrophage Stat6 activation in reducing tubular cell death (Fig. 7g, h). Similar to AS1517499 treatment, downregulating Stat6 expression with *Stat6* siRNA transfection in macrophages led to more TNFα production and tubular cell death (Fig. 7i–l). Next, in mouse models, p-Stat6 could be detected in tubule, T lymphocytes, and F4/80-staining-positive macrophages in UUO and IRI kidneys (Supplementary Fig. 6a, b and Fig. 7m). Much more p-Stat6 was detected in macrophages from MФ-PP2Acα^−/−^ fibrotic kidneys (Fig. 7m–o). Western blot analyses further confirmed this finding (Fig. 7p–s). Together, these data show that PP2Acα deficiency leads to Stat6 phosphorylation at T645 to reduce TNFα production and tubular cell death.

### Inhibition of PP2Acα with phendione attenuates renal fibrosis caused by UUO and IRI

**Fig. 8:**
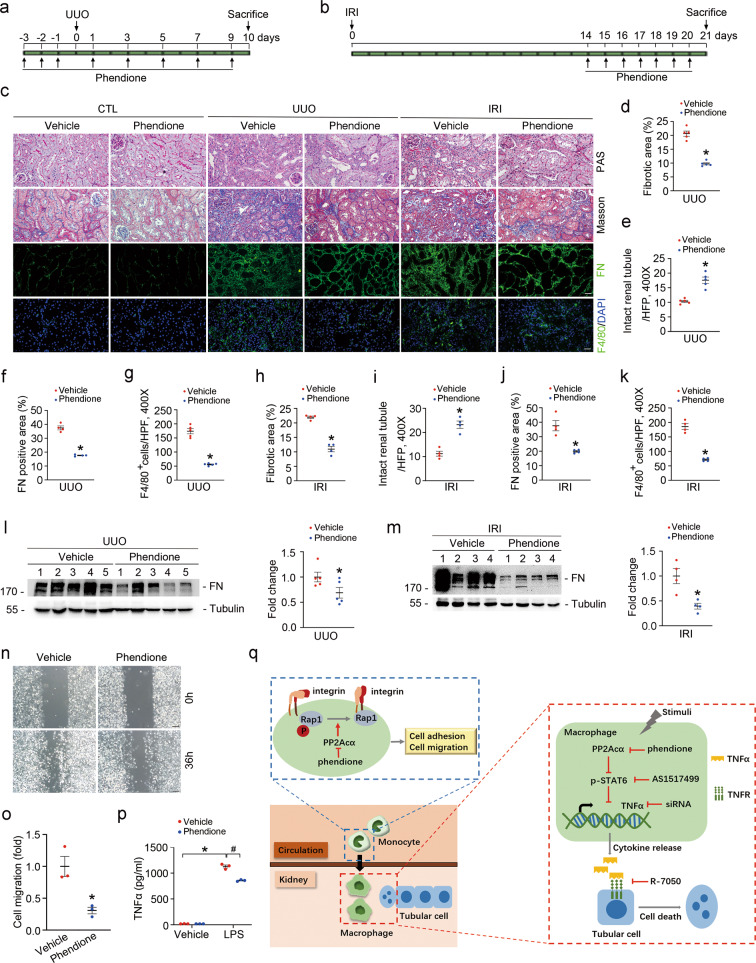
Inhibition of PP2Acα with phendione attenuates renal fibrosis in mice after UUO and IRI. **a**, **b** Strategy for surgery and phendione administration in mice as indicated. **c** Representative micrographs for PAS, Masson, FN, and F4/80 staining in kidney tissues. Scale bar, 20 μm. **d**–**g** Graphic presentation showing the fibrotic area (**d**), intact renal tubule (**e**), FN-staining-positive area (**f**) and F4/80-positive cells (**g**) in UUO kidney tissues among groups as indicated. *p < 0.05, *n* = 5. Data are presented as means ± SEM. **h**–**k** Graphic presentation showing the fibrotic area (**h**), intact renal tubule (**i**), FN-staining-positive area (**j**), and F4/80-staining-positive cells (**k**) in IRI kidney tissues among groups as indicated. *p < 0.05, *n* = 4. Data are presented as means ± SEM. **l** Western blot analyses (left) and quantitative analysis (right) for FN in UUO kidneys from Vehicle and phendione-treated mice. Numbers 1–5 indicate each individual animal within a given group. *p < 0.05, *n* = 5. Data are presented as means ± SEM. **m** Western blot analyses (left) and quantitative analysis (right) for FN in IRI kidneys from Vehicle and phendione-treated mice. Numbers 1–4 indicate each individual animal within a given group. *p < 0.05, *n* = 4. Data are presented as means ± SEM. **n**, **o** Representative images (**n**) and quantitative analysis (**o**) for wound healing test in cultured BMDMs from different groups as indicated. Scale bar, 100 μm. *p < 0.05, *n* = 3. Data are presented as means ± SEM. **p** ELISA analysis showing the protein abundance of TNFα in the cultural media of BMDMs. *p < 0.05, *n* = 3. ^#^*p* < 0.05, *n* = 3. Data are presented as means ± SEM. **q** Working model of this study.

To investigate whether pharmacologically blocking PP2Acα could attenuate kidney fibrosis, male CD1 mice subjected to UUO or IRI were treated with phendione, an inhibitor of PP2Acα by binding its catalytic cleft (Fig. 8a, b). Phendione treatment could markedly attenuate tubular atrophy, interstitial matrix deposition, and inflammatory cell infiltration in mice after UUO or IRI (Fig. 8c, d, f–h, j–m). Notably, the integrity of renal tubule was largely preserved after phendione treatment (Fig. 8c, e, i). In cultured BMDMs, phendione treatment decreased cell migration and TNFα production (Fig. 8n–p). Together, these results suggest that inhibition of PP2Acα with phendione diminishes macrophage migration and TNFα production, preserves tubular integrity, and attenuates kidney fibrosis in mice after UUO or IRI.

## Discussion

We report here that macrophage PP2Acα induction plays a crucial role in promoting kidney fibrosis through two independent mechanisms. First, PP2Acα induction in macrophages facilitates kidney tissue macrophage infiltration via Rap1-regulated migration. Second, macrophage PP2Acα induction promotes tubular cell death and kidney fibrosis through upregulating Stat6-mediated TNFα production in macrophages (Fig. 8q). In addition, we also demonstrated that targeting PP2Aca with phendione may be a promising strategy for attenuating kidney fibrosis.

PP2A, a ubiquitously expressed serine/threonine phosphatase accounting for a large fraction of phosphatase activity in eukaryotic cells, is composed of three subunits: structural A subunit (PP2A_A_), regulatory B subunit (PP2A_B_), and catalytic C subunit (PP2A_C_) [20]. PP2A_C_ has two isoforms: α and β. α isoform is about ten times more than β in kidney tissues [21, 22]. In this study, both PP2Acα and PP2Acβ were detected in the sham and fibrotic kidney tissues; however, only PP2Acα was consistently induced in macrophages from the fibrotic kidneys, suggesting PP2Acα but not PP2Acβ is crucial in regulating macrophage activation during kidney fibrosis. Sun et al. reported that mice with myeloid-specific deletion of PP2Acα developed more exuberant lung fibrosis in response to bleomycin [18], while Hou et al. and Deng et al. reported that targeting PP2A retarded kidney fibrosis, suggesting a pro-fibrotic role for PP2A in kidney diseases [23, 24]. Consistently, in this study, we found that ablation of PP2Acα in macrophages reduced UUO- or IRI-induced kidney fibrosis in mice. In addition, pharmacologically targeting PP2Acα with phendione could inhibit macrophage migration and TNFα production, thereby preserved tubular integrity and attenuated kidney fibrosis in mice after UUO or IRI. In this study, depletion of macrophage PP2Acα in mice largely reduced macrophage accumulation in the fibrotic kidneys. Compared to the control littermates, the population of peripheral monocytes/macrophages in the knockouts was reduced about 31%, whereas macrophage accumulation in MФ-PP2Acα^−/−^ fibrotic kidneys was reduced much more (~55%) compared to MФ-PP2Acα^+/+^ fibrotic kidneys. Since macrophage proliferation and survival were similar between PP2Acα^+/+^ and PP2Acα^−/−^ macrophages, while cell migration was largely inhibited in PP2Acα^−/−^ macrophages, we drew a conclusion that PP2Acα deficiency diminishes macrophage accumulation resulting from reduced macrophage infiltration.

The activity of PP2Ac may be regulated by modulating its phosphorylation and methylation status. DeGrande et al. report that PP2A catalytic, regulatory, and scaffolding units are tightly regulated at transcriptional, translational, and post-translational levels to tune myocyte function at base line and in disease. They found that the tyrosine phosphorylation of PP2Ac at Tyr307 was increased, whereas the methylation of PP2Ac at Leu309 was decreased in human heart failure samples and hydrogen hyperoxide-treated myocytes, which favor the inactivation of the holoenzyme [25]. Serum, TNFα, IL1, and EGF could stimulate the tyrosine phosphorylation of PP2A in fibroblasts [26–28]. CSF-1 could regulate the tyrosine phosphorylation of PP2A in myeloid cells [29]. In this study, we found that PP2Acα and methyl-PP2Ac were induced in kidney tissues from mice after UUO or IRI. Although LPS could largely upregulate PP2Acα expression and methylation (Supplementary Fig. 1), in macrophages sorted from the fibrotic kidneys, PP2Acα induction but not its methylation was detected compared to those in the spleen, suggesting that the transcriptional upregulation may play a major role for regulating macrophage PP2Acα during kidney fibrosis.

As a highly conserved serine/threonine phosphatase, PP2A is responsible for more than 90% of the protein dephosphorylation. It was reported that PKA-dependent phosphorylation of Rap1 on serine 179/180 regulates its membrane localization and cell migration [30]. In this study, we found that PP2Acα regulates macrophage infiltration through Rap1 based on the following evidences. First, deleting PP2Acα increased the serine/threonine phosphorylation of Rap1, reduced Rap1 activity and protein abundance, and inhibited macrophage spreading and migration in macrophages. Second, overexpression of Rap1bV12 could reverse the decreased cell migration caused by PP2Acα depletion. Besides phosphorylation, as a member of the Ras-like small GTP binding protein family, Rap1 activity is positively regulated by GEFs and negatively regulated by GAPs. Epac is considered as a major GEF for Rap1. However, in this study, we found that Epac1 abundance was not changed in macrophages with PP2Acα ablation or overexpression. In addition, downregulating Epac1 expression had little effect on macrophage motility. Therefore, it may be concluded that Epac1 does not mediate PP2Acα-regulated Rap1 function. Cell adhesion and migration are tightly regulated by integrins. Here, we found that ablation of PP2Acα led to increased Rap1 phosphorylation, decreased Rap1 activity, and its association with β2 integrin, as well as less integrin β2 clustering in macrophages. All data demonstrate a critical role for PP2Acα in regulating Rap1 and integrin activity.

Macrophages recruited to the injured site may produce large amounts of cytokines to modulate tubular cell fate [10, 13]. Studies have demonstrated that TNFα induction contributes to the development of multiple kidney diseases including cisplatin-induced renal damage, angiotensin II-induced glomerular damage, diabetic nephropathy, and obstruction-induced nephropathy [31–33]. In ischemia and obstructive-induced nephropathy, TNFα is identified as an important mediator of tubular cell apoptosis [34–36]. In this study, we found that ablation of PP2Acα in macrophages could reduce TNFα production. Tubular cell apoptosis was reduced and the integrity of renal tubule was preserved in MФ-PP2Acα^−/−^ fibrotic kidneys. Less tubular cell death was observed in primary cultured tubular cells co-cultured with LPS-treated PP2Acα^−/−^ macrophages. Furthermore, TNFα siRNA and R-7050 treatment significantly decreased tubular cell death caused by the CM of LPS-treated PP2Acα^+/+^ macrophages. Therefore, multiple evidence showed that less tubular cell death in MФ-PP2Acα^−/−^ fibrotic kidneys is due to downregulated TNFα expression in macrophages.

A network of signaling molecules, transcription factors, and post-transcriptional regulators modulate macrophage activation [37]. Stat6 signaling activation could inhibit macrophage M1 polarization by inhibiting Stat1 and Irf-5, which is essential for the production of cytokines including IL-12, IL-23, and TNFα [38–42]. It has been reported that PP2A rather than PP1 selectively regulates serine phosphorylation of Stat6 [43–46]. In this study, we found that ablation of PP2Acα could markedly induce the phosphorylation of Stat6 at T645. In addition, AS1517499 and Stat6 siRNA transfection reversed TNFα reduction in PP2Acα^−/−^ BMDMs. Therefore, it is conclusive that macrophage PP2Acα ablation attenuates macrophage TNFα production via Stat6.

In summary, this study demonstrated that macrophage ablation of PP2Acα ameliorates tubular cell apoptosis and renal fibrosis by reducing macrophage accumulation, macrophage activation, and TNFα production. Targeting macrophage PP2Acα may provide a useful strategy for retarding kidney tubulointerstitial fibrosis.

## Materials and methods

### Mice and animal models

Male C57BL/6 mice weighing approximately 18–20 g were acquired from the specific pathogen-free laboratory animal center of Nanjing Medical University. Mice were sacrificed and kidneys were harvested at different time points after UUO. For kidney IRI model, the left renal pedicle of the mouse was clamped for 30 min. The right kidneys were not removed. Mice were sacrificed and kidneys were harvested at different time points after IRI.

Mice expressing tamoxifen-inducible MerCreMer fusion protein under the control of macrophage-specific mouse Csf1r promoter (019098, FVB-Tg (Csf1r-cre/Esr1*)) were ordered from Jackson Laboratories (Bar Harbor, ME). FVB-Tg (Csf1r-cre/Esr1*) mice were crossed with C57BL/6J mice for eight generations to get Csf1r-Cre transgenic mice on C57BL/6J background. Homozygous PP2Acα floxed mice were kindly provided by Dr Chaojun Li from Nanjing University [47]. By mating PP2Acα floxed mice with Csf1r-Cre/Esr1* transgenic mice, mice that were heterozygous for the PP2Acα floxed allele were generated (genotype: Csf1r-Cre^+/^^−^, PP2Acα^fl/wt^). These mice were crossbred with homozygous PP2Acα floxed mice (genotype: PP2Acα^fl/fl^) to generate offspring with different littermates (Csf1r-Cre^+/^^−^, PP2Acα^fl/fl^; Csf1r-Cre^+/^^−^, PP2Acα^fl/wt^; Csf1r-Cre^−/−^, PP2Acα^fl/wt^; Csf1r-Cre^−/−^, PP2Acα^fl/fl^). Mice with genotyping Csf1r-Cre^+/^^−^, PP2Acα^fl/fl^, and the same gender littermates with genotyping Csf1r-Cre^−/−^, PP2Acα^fl/fl^ were utilized. Genotyping was performed by PCR assay using DNA extracted from the mouse tail. Csf1r-Cre^+/^^−^, PP2Acα^fl/fl^ mice and control littermates were subjected to UUO operation and intraperitoneally injected with tamoxifen (T5648; Sigma-Aldrich, St. Louis, MO) at 50 mg/kg at day 3 and 25 mg/kg at day 4 and 5 after UUO, respectively. Csf1r-Cre^+/^^−^, PP2Acα^fl/fl^ mice and control littermates were subjected to IRI operation and intraperitoneally injected with tamoxifen at 25 mg/kg for 7 consecutive days starting at day 7 after IRI.

Male CD1 mice weighing 20–25 g acquired from the specific pathogen-free laboratory animal center of Nanjing Medical University were subjected to IRI and intraperitoneally injected with phendione at 1 mg/kg for 7 consecutive days starting at day 14 and sacrificed at day 21 after IRI. For UUO, male CD1 mice were injected with phendione (cat: 496383, Sigma-Aldrich) at 1 mg/kg for 3 consecutive days and then subjected to UUO operation. After surgery, mice were injected with phendione at 1 mg/kg every other day and sacrificed at day 10.

All animals were housed in the specific pathogen-free laboratory animal center of Nanjing Medical University according to the guidelines of the Institutional Animal Care and Use Committee at Nanjing Medical University.

### Cell culture and treatment

BMDMs were isolated as previously described [48, 49]. BMDMs were cultured in DMEM containing 10% (v/v) FBS (Invitrogen, Grand Island, NY), 10 ng/ml mouse M-CSF (cat: 416-ML-050; R&D Systems, Minneapolis, MN), and 1% (v/v) antibiotics (100 U/ml penicillin) for 9 days. The medium was changed every other day. To generate BMDMs with PP2Acα deletion, BMDMs from Csf1r-Cre^+/−^, PP2Acα^fl/fl^ mice were treated with 1 mM 4-OHT (H6278; Sigma-Aldrich) at the beginning of the culture. BMDMs from Csf1r-Cre^−/−^, PP2Acα^fl/fl^ mice were treated with 4-OHT as control. On day 9, BMDMs were cultured with serum-free medium and treated with LPS (100 ng/µl) (*Escherichia coli* 0111: B4, Sigma-Aldrich). To inhibit Stat6 phosphorylation, BMDMs were treated with AS1517499 (cat: HY-100614, MCE, USA) for 30 min, then stimulated with LPS. To block TNFα signaling, primary tubular epithelial cells were treated with R-7050 (cat: HY-110203, MCE) for 30 min, then treated with macrophage CM. To inhibit PP2Acα activity, BMDMs were treated with phendione.

### Small interfering RNA (siRNA) transfection

siRNAs specific for mouse *Tnfα*, *Epac1*, *Itgb2*, and *Stat6*, respectively, were ordered from Shanghai Integrated Biotech Solutions Co., Ltd. The target sequence of the murine *Tnfα* is GCAUGGAUCUCAAAGACAACC; of the murine *Epac1* is GCUUCAACGAGCUGCAGUACU; of the murine *Itgb2* is GGUAUGACGCUGCAGACUAUC; of the murine *Stat6* is GAUGCUUUCUGUUACAACAUG. BMDMs were transfected with siRNAs using Lipofectamine 3000 (Invitrogen, Grand Island, NY) according to the manufacturer’s instruction.

### Western blot assay

BMDMs were lysed in 1×SDS sample buffer. Kidneys were lysed with RIPA buffer containing 1% NP-40, 0.1% SDS, 100 mg/ml PMSF, 1% protease inhibitor cocktail, and 1% phosphatase I and II inhibitor cocktail (Sigma-Aldrich) on ice. The supernatants were collected after centrifugation at 16,000 g at 4 °C for 30 min. Protein concentration was determined by bicinchoninic acid protein assay (BCA Protein Assay Kit, Pierce Thermo-Scientific, Rockford, IL) according to the manufacturer’s instruction. The primary antibodies were anti-PP2Ac (cat: 2038, Cell Signaling Technology, Boston, MA, USA, 1:1000), anti-PP2Acα (cat: ab106262, Abcam, Cambridge, UK, 1:1000), anti-PP2Acβ (cat: ab168371, Abcam, 1:1000), anti-methyl-PP2Ac (L309) (cat: ab66597, Abcam, 1:1000), anti-Itgb2 (cat: ab119830, Abcam, 1:1000), anti-Epac1 (cat: ab124162, Abcam, 1:1000), anti-FN (cat: F3648, Sigma-Aldrich, 1:10000), anti-Stat6 (cat: ab32520, Abcam, 1:1000), anti-p-Stat6 (T645) (cat: BS4186, Bioworld Technology, Nanjing, China, 1:1000), anti-Rap1a/b (cat: 4938, Cell Signaling Technology, 1:1000), anti-tubulin (cat: sc53646, Santa Cruz Biotechnology, 1:10000), and anti-GAPDH (cat: FL-335, Santa Cruz Biotechnology, 1:5000). Quantification was performed by measuring the intensity of the signals with the aid of the National Institutes of Health ImageJ software package.

### Immunoprecipitation assay

BMDMs were lysed with lysis buffer (Beyotime, Shanghai, China) containing 1% protease inhibitor cocktail and 1% phosphatase I and II inhibitor cocktail (Sigma-Aldrich) on ice. The supernatants were collected after centrifugation at 16,000 g at 4 °C for 15 min. Protein concentration was determined by BCA Protein Assay Kit (Pierce Thermo-Scientific) according to the manufacturer’s instruction. An equal amount of protein (1 mg) was incubated overnight at 4 °C with anti-phospho-(Ser/Thr) (cat: ab17464, Abcam, Cambridge, UK, 1:100) or anti-Itgb2 (cat: ab119830, Abcam, 1:250) antibody. After incubating with protein-A/G PLUS-Agarose beads (Santa Cruz, Dallas, TX) for 3 h, the beads were collected for further analysis.

### Measurement of Rap1 activity in BMDMs

Cells were harvested after being washed two times by cold PBS, and extracted in an ice-cold 50 mM HEPES-based buffer (PH 7.4) containing 10 mM MgCl2, 150 mM NaCl, 1% Nonidet P-40, 0.5 mM phenylmethylsulfonyl fluoride, protease inhibitor, and phosphatase inhibitors. The total Rap1 in the supernatants was precipitated with 3 μg of anti-Rap1a/b antibody. GTP was converted to ATP using NDP kinase and ADP with the resulting ATP measured in the luciferase/luciferin system with the ATP Bioluminescent Assay Kit (product number: FL-AA; Sigma-Aldrich). The sum of GTP plus GDP was measured by converting GDP to GTP using pyruvate kinase and phosphoenolpyruvate and then the total GTP, representing the sum of GDP plus GTP, was measured as described above. The result was presented as the ratio of GTP to GTP plus GDP [50].

### Real-time qRT-PCR assay

Total RNA was extracted using Trizol reagent (cat: 15596018, Invitrogen) according to the manufacturer’s instruction. cDNA was synthesized using 1 μg of total RNA, ReverTra Ace (cat: R111-02, Vazyme, Nanjing, China), and oligo (dT)12–18 primers according to the manufacturer’s protocol. Gene expression was measured by real-time qRT-PCR (cat: Q141–02, Vazyme) and 7300 real-time PCR system (Applied Biosystems, Foster City, CA, USA). The relative amount of mRNA to internal control was calculated using the equation 2 ∆CT, in which ∆CT = CTgene – CTcontrol.

### Histology and immunohistochemical staining

Mouse kidney samples were fixed in 10% neutral-formalin, embedded in paraffin. Three-μm thickness sections were stained with periodic acid–Schiff, Masson, and Sirius red. Human kidney biopsies diagnosed with TIN, DN, and FSGS were from the Second Affiliated Hospital of Nanjing Medical University. The slides were immune-stained with antibodies against CD68 (cat: MO876, Dako, Denmark, 1:200) and PP2Ac (cat: 2038, Cell Signaling Technology, 1:50). Slides were viewed under OLYMPUS DP74 microscope equipped with a digital camera.

### Immunofluorescence staining

Kidney cryosections at 3-μm thickness were fixed with 4% paraformaldehyde for 15 min followed by permeabilization with 0.2% Triton X-100 in 1×PBS for 5 min at room temperature. After blocking with 2% donkey serum for 60 min, the slides were immune-stained with the following antibodies: anti-FN (cat: F3648, Sigma-Aldrich), anti-F4/80 (cat: 14–4801, eBioscience, San Diego, CA, USA), anti-CD3 (cat: 555273, BD Pharmingen, New Jersey, USA), anti-PP2A C subunit (cat: 2038, Cell Signaling Technology), anti-Stat6 (phospho-T645) (cat: BS4186, Bioworld Technology), anti-TNFα (cat: BS1857, Bioworld Technology), anti-Laminin (cat: ab44941, Abcam, 1:100), anti-Itgb2 (cat: ab119830, Abcam, 1:200), and anti-CD68 (cat:MO876, Dako). Tissues were stained with DAPI to visualize the nuclei. Slides were viewed with an OLYMPUS DP74 and BX53 Epifluorescence microscope equipped with a digital camera. The number of F4/80-positive and CD3-positive macrophages was counted from ten randomly selected fields in the cortical area for each sample under microscope (400×), and an average number of positive cells for each section was calculated.

### Kidney monocyte/macrophage enrichment

After perfusion with cold 1xPBS, kidneys were removed and minced into fragments, then digested in DMEM containing 1 mg/ml collagenase (cat: 17018–029, Gibco, New York, USA) and 0.1 mg/ml DNase (cat: 10104159001, Roche, Basel, Switzerland) using Octo Dissociator with Heaters (gentleMACS, Miltenyi Biotec, Germany). The fragments were filtered through 40-μm mesh (Falcon, BD Biosciences, New Jersey, USA) to get single-cell suspension. Macrophages were enriched from the single-cell suspension with PE-anti CD115 antibody (cat: 135505, Biolegend, San Diego, USA), PE Microbeads (cat: #17666, Stemcell, Canada), and DYNAL bead separations (Invitrogen) according to the manufacturer’s instruction.

### Terminal deoxynucleotidyl transferase-mediated dUTP nick-end labeling (TUNEL) staining

TUNEL staining was employed to determine apoptotic cells by using the apoptosis detection system (Promega, Madison, WI, USA).

### Propidium iodide (PI) staining

PI staining was employed to determine dead cells (cat: E-CK-A161, Elabscience, China) according to the manufacturer’s instruction.

### TNFα ELISA assay

The kidneys were lysed with cold 1×PBS buffer containing 100 mg/ml PMSF, 1% protease inhibitor cocktail, and 1% phosphatase I and II inhibitor cocktail (Sigma-Aldrich) on ice. The supernatants were collected after centrifugation at 5000 g at 4 °C for 5 min. Protein concentration was determined by BCA Protein Assay Kit (Pierce Thermo-Scientific) and soluble TNFα was detected with the Mouse TNFα ELISA kit (cat: CEK1783, Bioworld Technology) according to the manufacturer’s instructions.

### Flow cytometry

Peripheral blood from MФ-PP2Acα^+/+^ mice and MФ-PP2Acα^−/−^ mice was collected with EDTA as anticoagulant. After blocking with CD16/32 (cat: 101330, Biolegend) for 30 min, blood samples were incubated with PE-anti-Ly6g (cat: 83122-60-25, BioGems, Westlake Village, CA, USA), FITC-anti-Ly6c (cat: 553104, BD Biosciences, New Jersey, USA), and PerCP-anti-CD11b (cat: 101230, Biolegend, San Diego, USA) for 20 min. Then the red blood cells were lysed with RCLB. After centrifugation at 450 g for 5 min, cells were detected and analyzed with BD Canto II Flow Cytometer and the FlowJo software.

### Statistical analyses

All data examined are presented as mean ± SEM. Statistical analyses of the data were performed using SigmaStat software (Jandel Scientific Software, San Rafael, CA, USA). Comparison between groups was made using one-way ANOVA followed by the Student–Newman–Keuls test. Paired or unpaired *t* test was used to compare two groups. A *p* value of 0.05 or less was considered statistically significant.

## Supplementary information

Supplemental Figures
