# Supplementary material for: PP2Acα promotes macrophage accumulation and activation to exacerbate tubular cell death and kidney fibrosis through activating Rap1 and TNFα production
Source: Cell Death Differ. 2021 May 1;28(9):2728–44. doi: 10.1038/s41418-021-00780-5 (PMC8408198; doi:10.1038/s41418-021-00780-5)

Supplemental Figure 1. LPS treatment upregulates PP2Acα expression and methylation in BMDMs.

(a) Western blot analyses showing the abundance of PP2Acα and methyl-PP2Acα (L309) in BMDMs after LPS-treatment.

(b) Real-time qRT-PCR analysis showing the mRNA abundance of PP2Acα in LPS-treated BMDMs. *$p<0.05$, n=3. Data are presented as means ± SEM.

Supplemental Figure 2. PP2Acα ablation has little effect on macrophage proliferation and death in the fibrotic kidneys.

(a) Representative micrographs for TUNEL and F4/80 co-staining in UUO and IRI kidneys. Scale bar, 10μm.

(b, c) Representative images (b) and quantitative determination (c) showing Ki67 staining positive BMDMs among different groups as indicated. Scale bar, 10μm. n=4. Data are presented as means ± SEM.

Supplemental Figure 3. Monocyte/macrophage population in the peripheral blood of MΦ-PP2Acα$^{+/+}$ and MΦ-PP2Acα$^{-/-}$ mice.

(a) Representative images for Ly6g-negative, Ly6c and CD11b-positive monocytes/macrophages in the peripheral blood of MΦ-PP2Acα$^{+/+}$ and MΦ-PP2Acα$^{-/-}$ mice.

(b) Quantitative analysis for Ly6g-negative, Ly6c and CD11b-positive monocytes/macrophages in the peripheral blood of MΦ-PP2Acα$^{+/+}$ and

MΦ-PP2Acα$^{-/-}$ mice. *p<0.05, n=4. Data are presented as means ± SEM.

Supplemental Figure 4. Epac1 is dispensable for PP2Acα-regulated macrophage migration.

(a) Western blot analyses showing Epac1 in PP2Acα$^{+/+}$ and PP2Acα$^{-/-}$ BMDMs.

(b) Western blot analyses showing Epac1 in PP2Acα-overexpressed BMDMs.

(c) Real-time qRT-PCR analysis showing the mRNA abundance of *Epac1* in scramble siRNA and Epac1 siRNA-transfected BMDMs. *p<0.05, n=3. Data are presented as means ± SEM.

(d, e) Representative images (d) and quantitative analysis (e) for wound healing test in cultured BMDMs from different groups as indicated. Scale bar, 100μm. *p<0.05, n=3. #p<0.05, n=3. Data are presented as means ± SEM.

Supplemental Figure 5. Inflammatory cytokine expression in kidneys.

(a) Real-time qRT-PCR analysis showing the mRNA abundance of *Tnfα, Il-1β, Il-6, Mcp1* and *Rantes* in the control kidneys from MΦ-PP2Acα$^{+/+}$ and MΦ-PP2Acα$^{-/-}$ mice. n=4. Data are presented as means ± SEM.

(b) Representative immune staining images showing the induction of TNFα in CD3 staining positive T cells within the kidneys after UUO or IRI. White arrows indicate co-staining positive cells. Scale bar, 10μm.

(c) Representative immune staining images showing the induction of TNFα in renal tubular cells within the kidneys after UUO or IRI. White arrows indicate co-staining positive cells. Scale bar, 10μm.

Supplemental Figure 6. p-Stat6 (T645) induction in the fibrotic kidneys.

(a) Representative immune staining images showing the induction of p-Stat6 (T645) in tubular cells within the kidneys after UUO or IRI. White arrows indicate p-Stat6 staining positive tubule. Scale bar, 10μm.

(b) Representative immune staining images showing the induction of p-Stat6 (T645) in CD3 staining positive T cells within the kidneys after UUO or IRI. White arrows indicate p-Stat6 staining positive T cells. Scale bar, 10μm.

Supplemental Fig 1

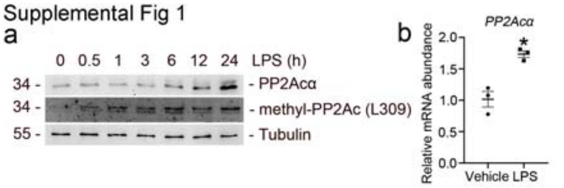

Supplemental Fig 2

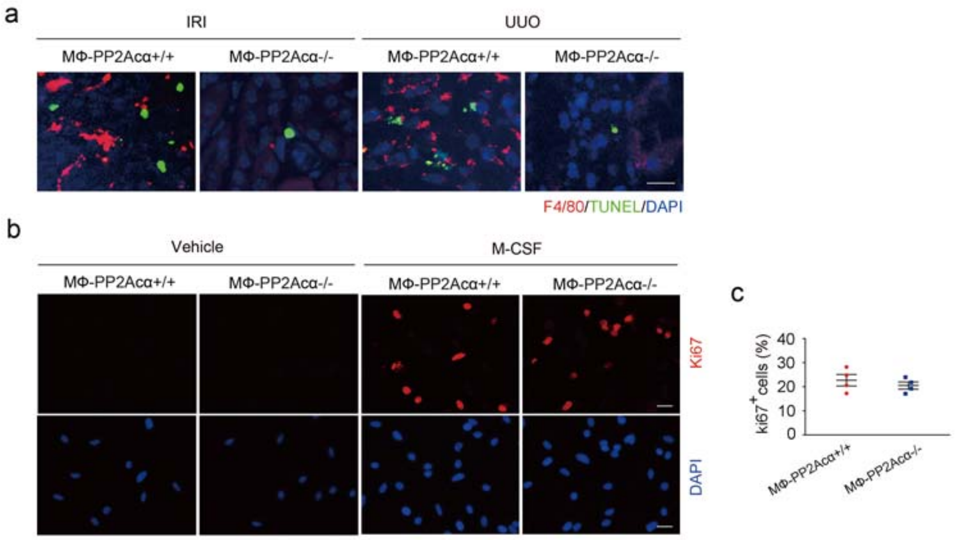

Supplemental Fig 3

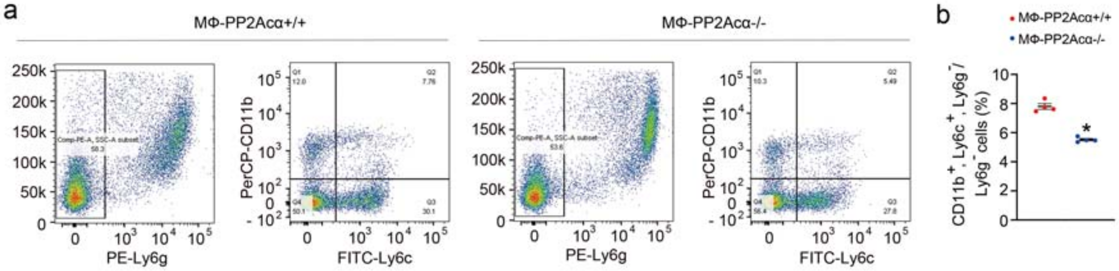

Supplemental Fig 4

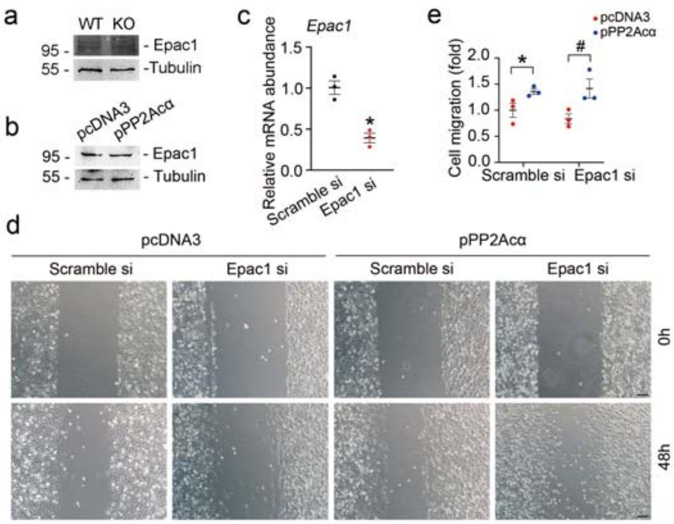

Supplemental Fig 5

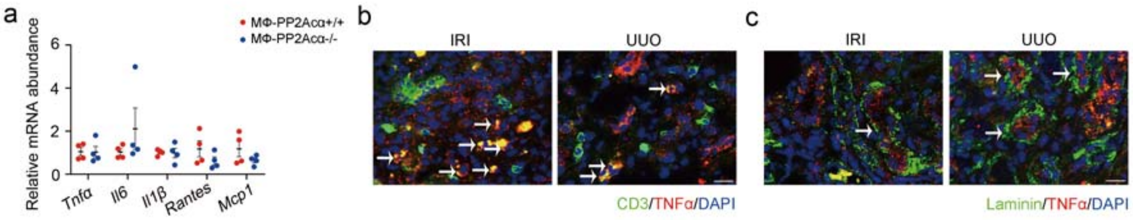

Supplemental Fig 6

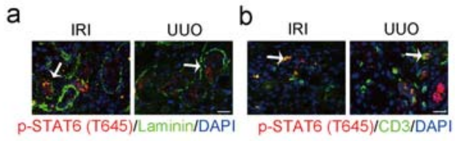

Supplement: Supplementary file 1 — Supplemental Figures [file 41418_2021_780_MOESM1_ESM.pdf]
